# Supplementary figures and images for: A point mutation decouples the lipid transfer activities of microsomal triglyceride transfer protein
Source: PLoS Genet. 2020 Aug 6;16(8):e1008941. doi: 10.1371/journal.pgen.1008941 (PMC7444587; doi:10.1371/journal.pgen.1008941)

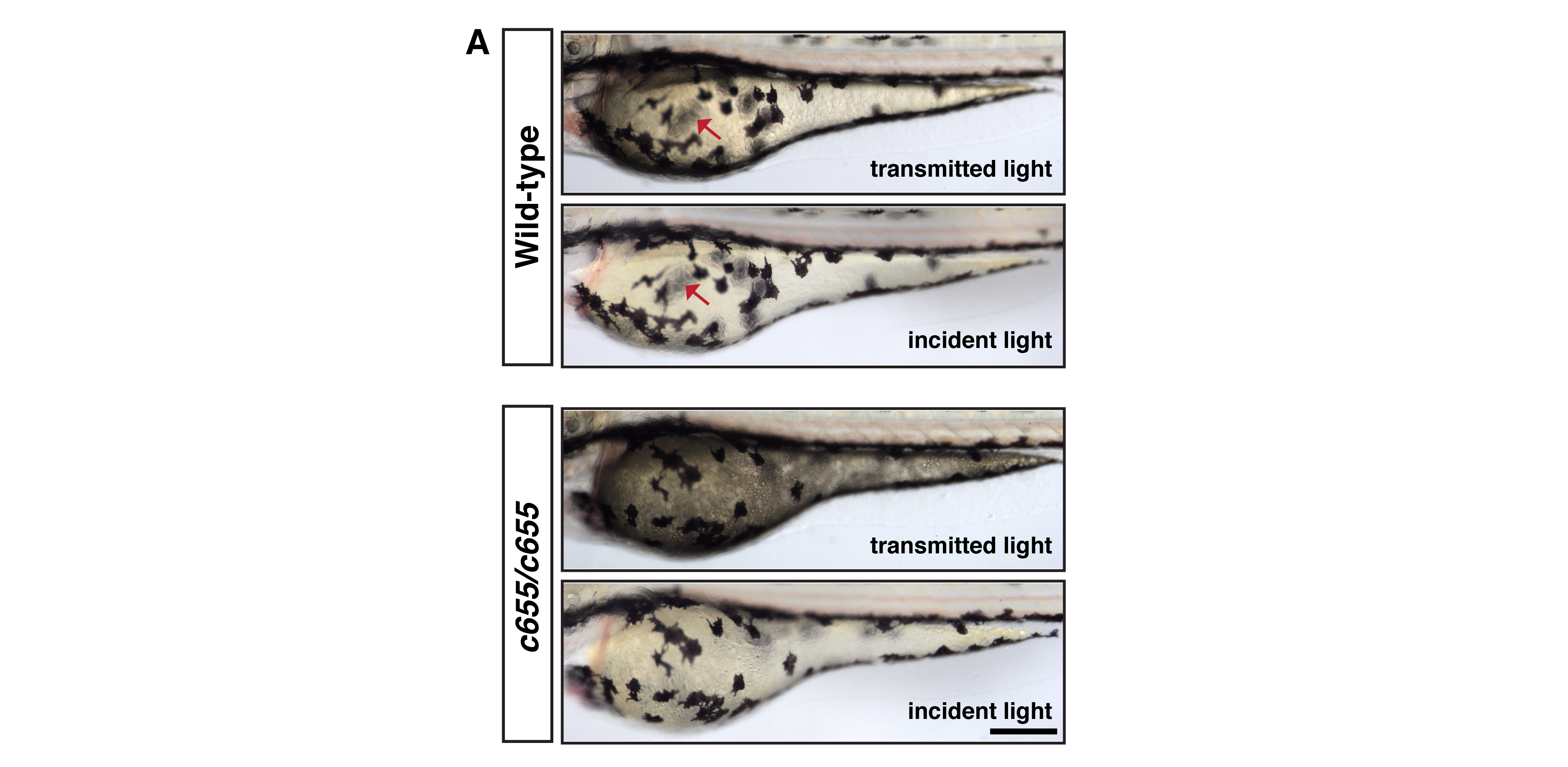

Supplement: S1 Fig — (A) Wild-type and mttpc655/c655 mutant embryos were imaged at 3 dpf using either transmitted light (illumination below the fish) or incident light (illumination from above the fish). The wild-type embryos are translucent; the pigment cells on the opposite side of the embryo (red arrow) are visible through the yolk with both light sources. The yolk is opaque in the mutants; it appears dark with transmitted light and off-white with incident light. Pigment cells on the opposite side of the embryo are barely visible in mutant embryos, regardless of light source. Scale = 200 μM. (TIF) [file pgen.1008941.s005.tif]

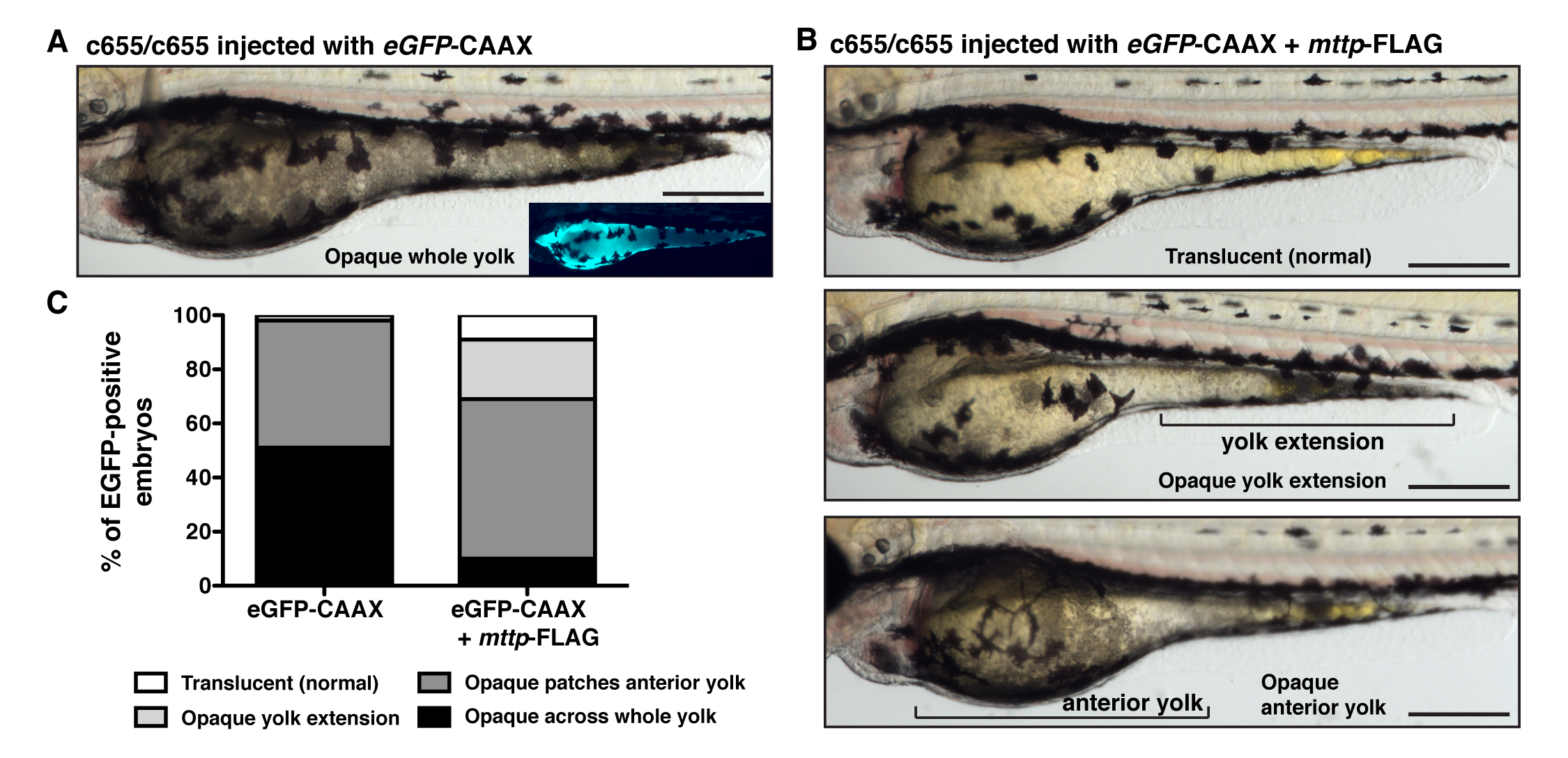

Supplement: S2 Fig — One-cell stage mttpc655/c655 embryos were co-injected with CMV: mttp-FLAG and the CMV: eGFP-CAAX plasmid, or CMV: eGFP-CAAX alone as a control. Embryos expressing eGFP-CAAX in the YSL were imaged at 3 dpf, and images were scored for the degree of yolk opacity by a lab member who was blinded to the genotype of the fish. (A) Representative image of an mttpc655/c655 mutant embryo expressing eGFP-CAAX in the YSL and a fully opaque yolk. (B) Examples of injected embryos with varying degrees of yolk opacity (normal translucent yolk, opaque region in the yolk extension, opaque patches in the anterior yolk with or without opaque yolk extension). (C) Images were binned into the four noted categories of yolk opacity. Results represent pooled data from 3 independent experiments, n = 91 control and 102 Mtp-FLAG eGFP-positive embryos total. Chi-square test, p < 0.001. Scale = 500 μM. (TIF) [file pgen.1008941.s006.tif]

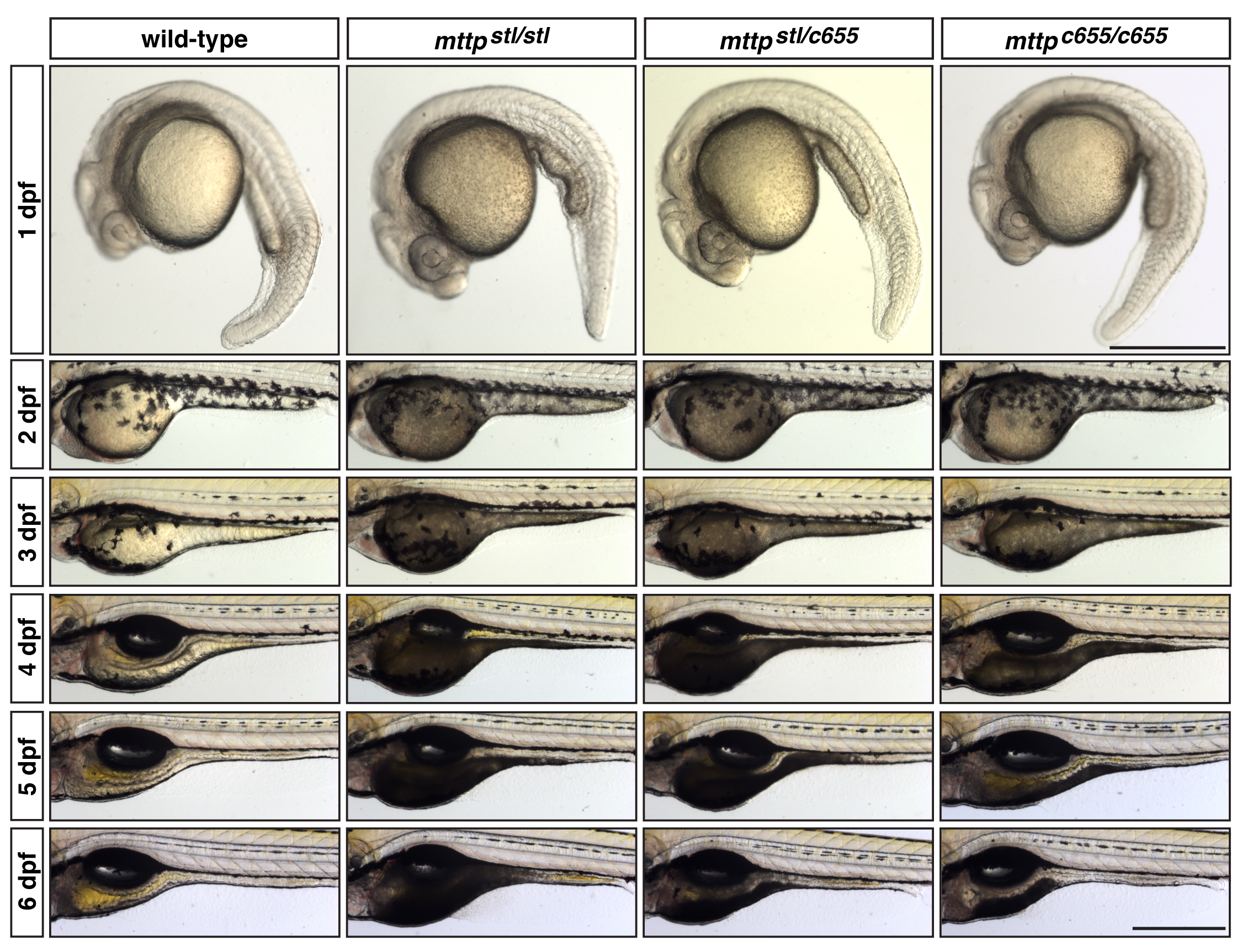

Supplement: S3 Fig — Representative images of wild-type, mttpstl/stl, mttpc655/c655 and trans-heterozygous mttp stl/c655 mutants from 1 dpf to 6 dpf. The mttpstl/stl mutants are visibly opaque at 1 dpf and the area of opacity is retained for longer than in mttpstl/c655 or mttpc655/c655 mutants. Images at 3 dpf are the same fish shown in Fig 1. Scale = 500 μM. (TIF) [file pgen.1008941.s007.tif]

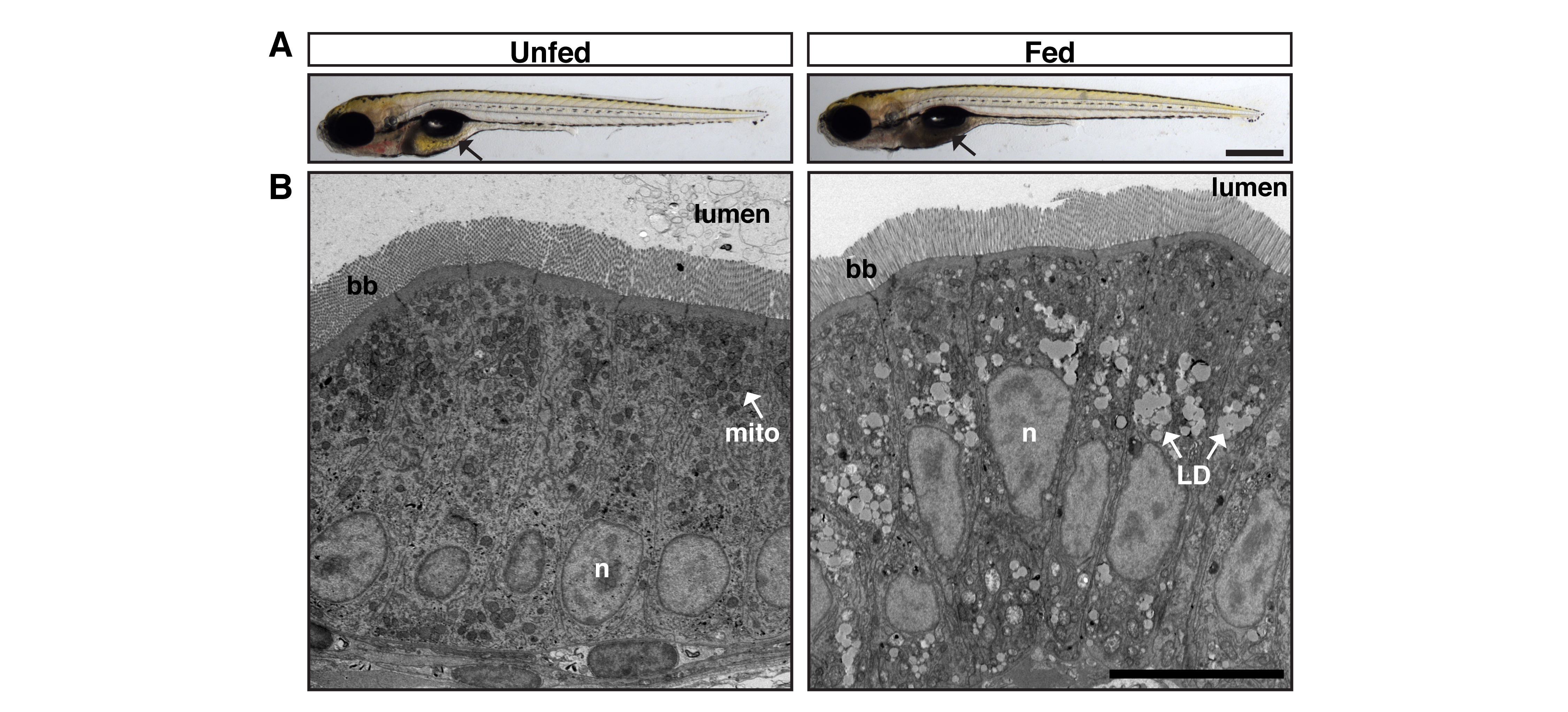

Supplement: S4 Fig — (A) Wild-type fish at 6 dpf were fed a high-fat meal for 1 h, as described previously [57]. Unfed fish have translucent intestines (black arrow, left) when imaged with transmitted light, whereas fed fish have opaque intestines (black arrow, right). Scale = 500 μM. (B) Electron microscopy following a 1 h high-fat feed reveals an accumulation of cytoplasmic lipid droplets in the intestinal enterocytes. By scattering light and blocking light transmission through the intestine, the accumulation of cytoplasmic lipid droplets causes the intestine to appear opaque. Nucleus (n), mitochondria (mito), brush border (bb), lipid droplet (LD). Scale = 10 μM. (TIF) [file pgen.1008941.s008.tif]

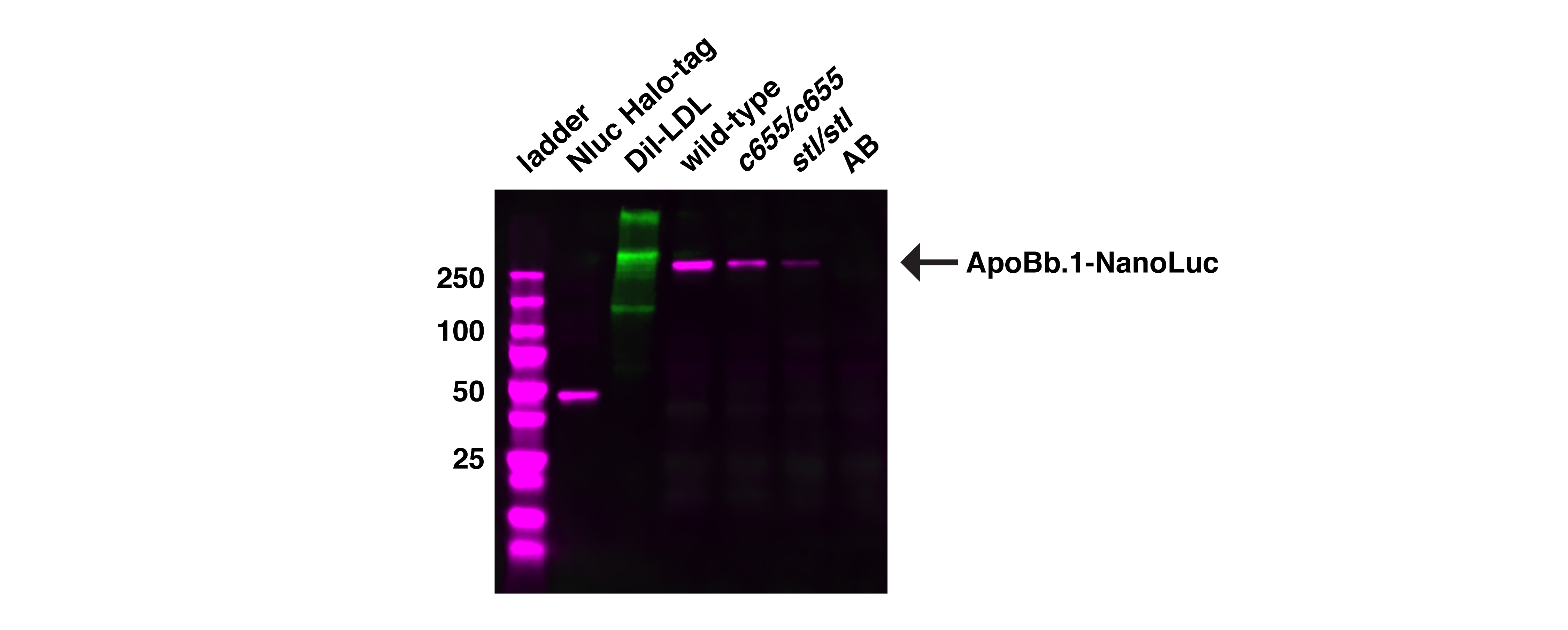

Supplement: S5 Fig — Representative immunoblot for the NanoLuc reporter in wild-type and mttp mutant zebrafish embryos. The NanoLuc reporter is fused to the C-terminus of the zebrafish apoBb.1 gene. Lanes represent lysate from 10 pooled 3 dpf mttp wild-type, mttpc655/c655, and mttpstl/stl mutant embryos, as well as wild-type AB embryos that do not carry the NanoLuc reporter. Purified Halo-tagged NanoLuc protein (Nluc Halo-tag, ~54 kDa) was used as a positive control for NanoLuc and DiI-LDL was used to mark the migration of APOB. Blot was probed simultaneously for NanoLuc (magenta) and Human APOB (green). The ApoBb.1-NanoLuc is exclusively detected as a high molecular weight band (>250 kDa) corresponding to the migration of human APOB. Note that the APOB antibody does not recognize zebrafish ApoB. (TIF) [file pgen.1008941.s009.tif]

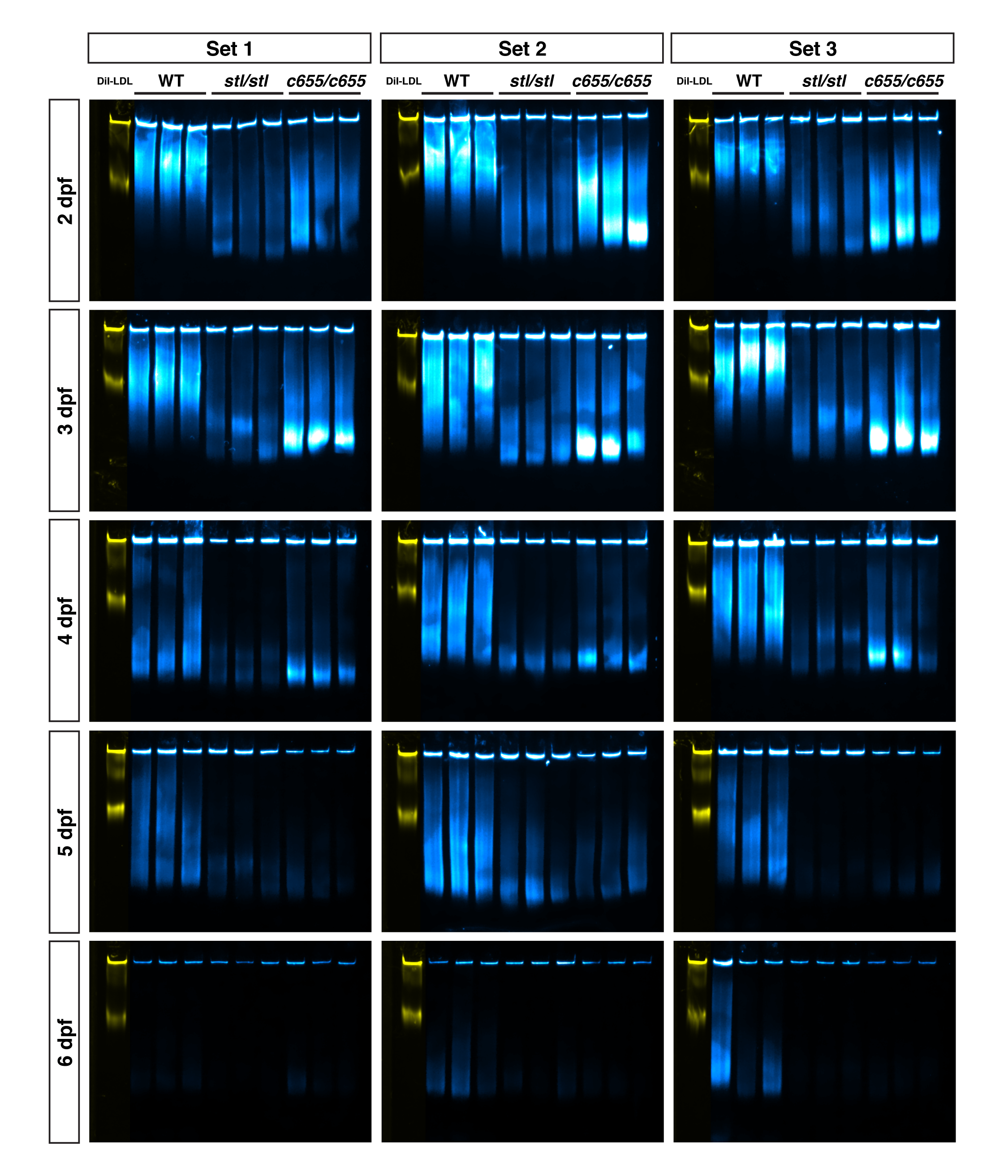

Supplement: S6 Fig — Original gels corresponding to the data in Fig 3D. Each gel shows a composite image of the fluorescent DiI-LDL migration standard (yellow) and LipoGlo emission chemiluminescent exposure (blue) from WT, mttpstl/stl, and mttpc655/c655 fish. Gels were analyzed as detailed in [48] and lipoprotein particles were binned into four classes based on migration relative to the DiI-LDL standard, including zero mobility (ZM), and three classes of serum B-lps (VLDL, IDL and LDL). (TIF) [file pgen.1008941.s010.tif]

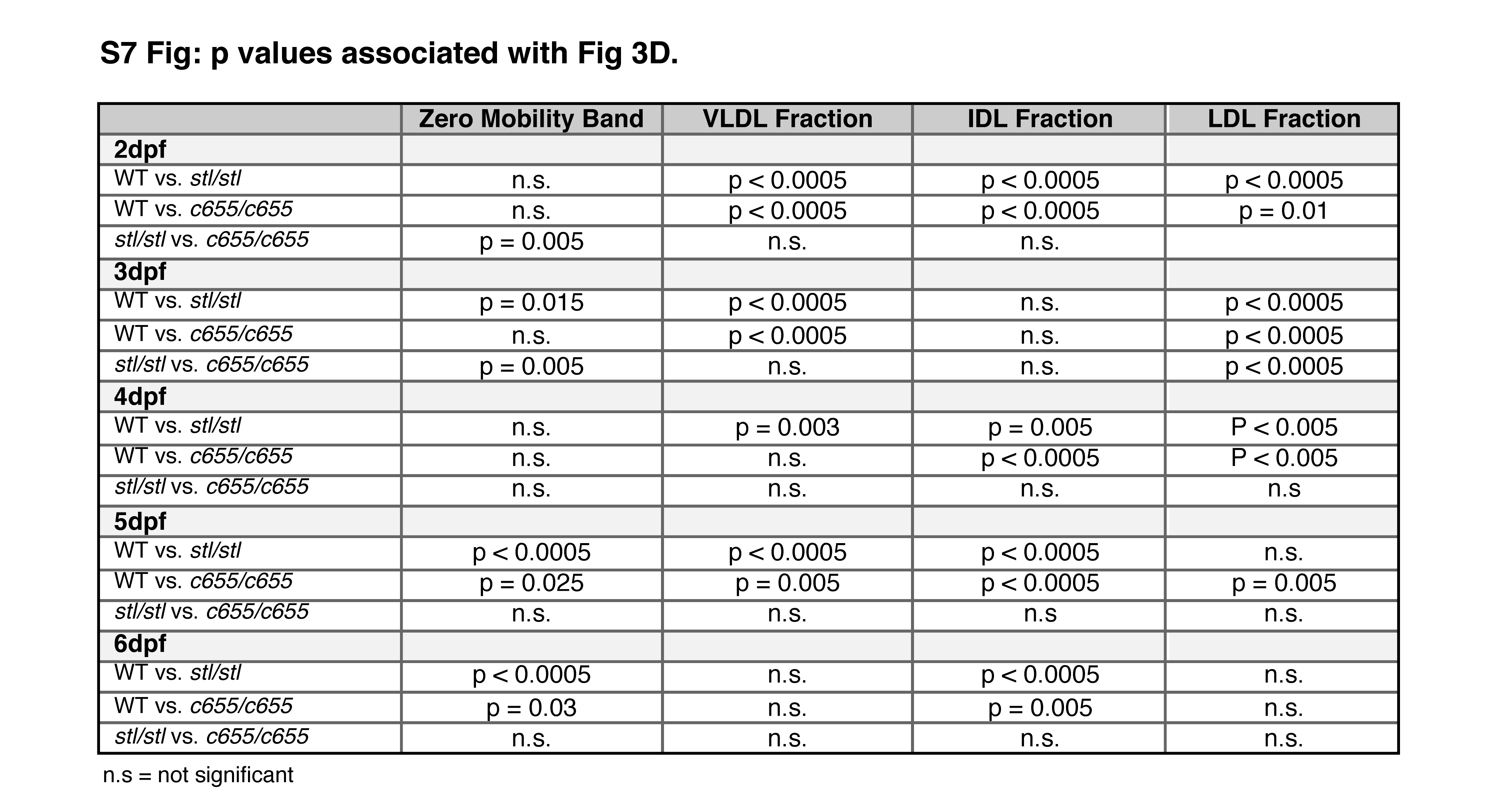

Supplement: S7 Fig — (TIF) [file pgen.1008941.s011.tif]

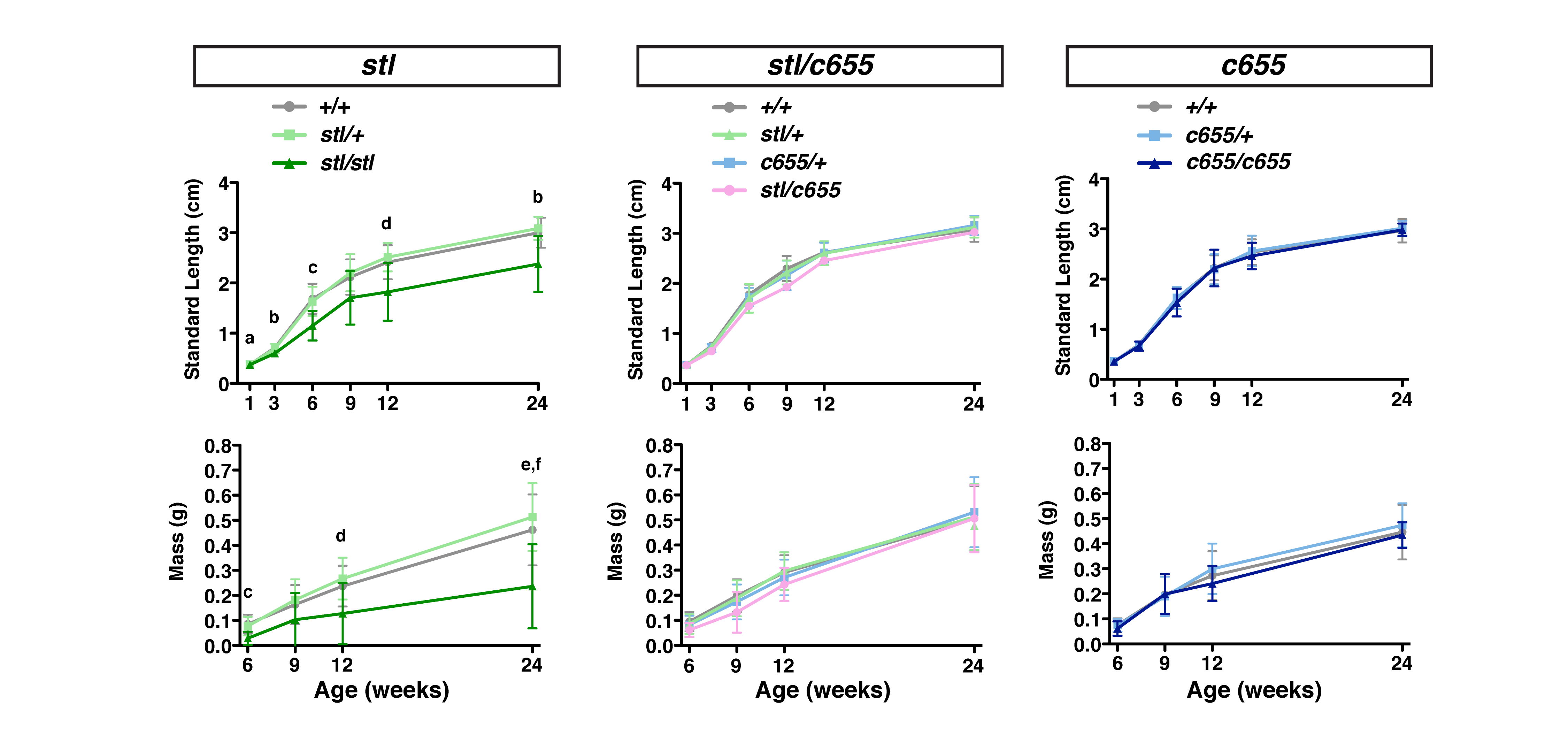

Supplement: S8 Fig — Results are representative of pooled data from two independent experiments, n = 7–80 fish/genotype/time-point, mean +/- SD. Significance was determined with a Robust ANOVA and Games-Howell post-hoc tests were used to make pair-wise comparisons at each time point. Using a Bonferroni correction, p-values were adjusted to control for multiple comparisons (6 length or 4 mass comparisons), a: stl/+ vs. stl/stl, p < 0.01, b: +/+ vs. stl/stl and stl/+ vs. stl/stl, p < 0.05, c: +/+ vs. stl/stl and stl/+ vs. stl/stl, p < 0.001, d: stl/+ vs. stl/stl, p < 0.05, e: +/+ vs. stl/stl, p < 0.01, f: stl/+ vs. stl/stl, p < 0.001. (TIF) [file pgen.1008941.s012.tif]

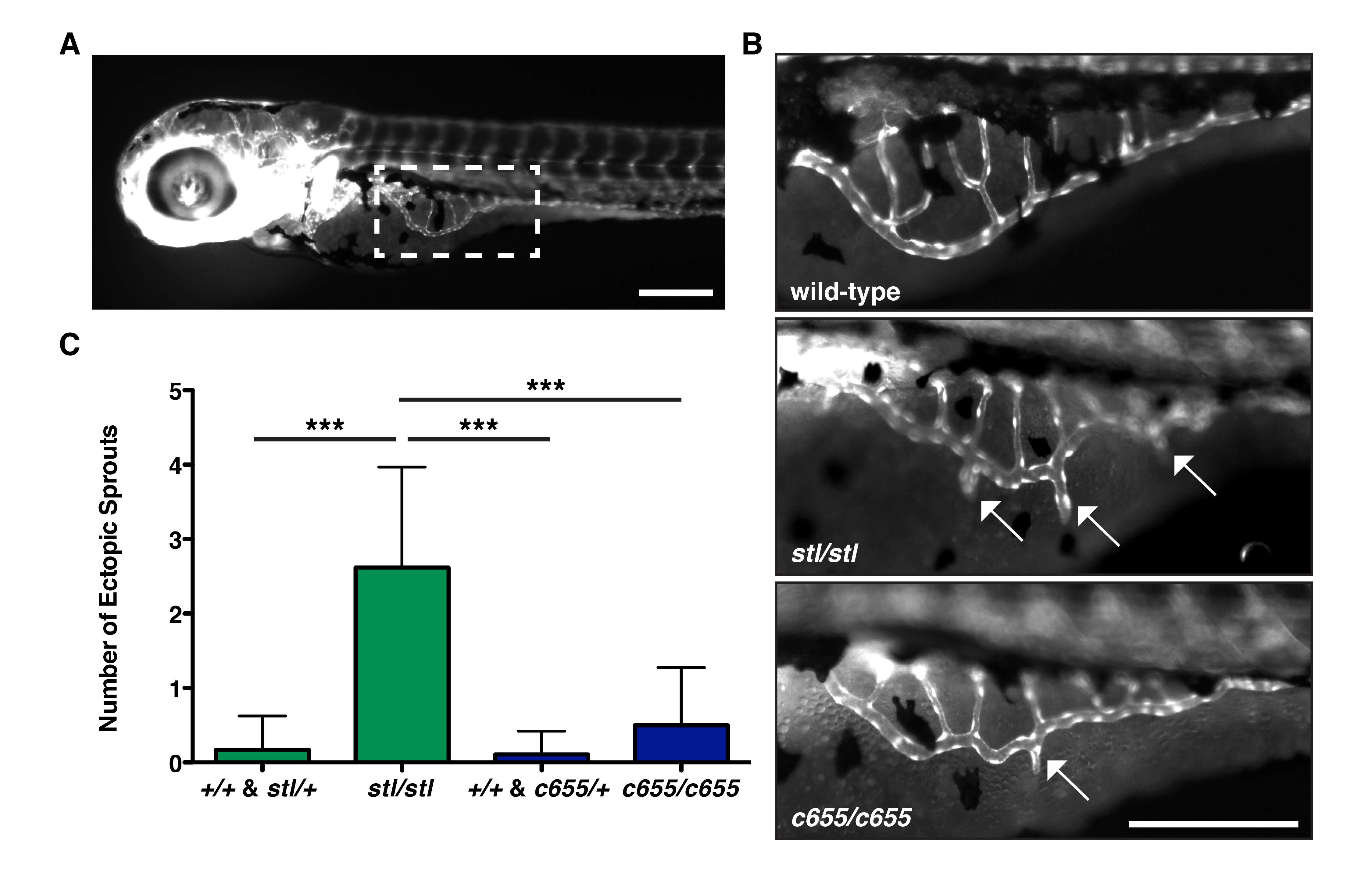

Supplement: S9 Fig — (A) The developing vasculature is visualized in the Tg(fli:eGFP)y1 transgenic zebrafish line [97]. The subintestinal vessels (boxed region) grow bilaterally onto the dorsolateral surface of the yolk sac. Scale = 200 μM. (B) Representative wide-field images of Tg(fli:eGFP)y1 in wild-type, mttpstl/stl, or mttpc655/c655 embryos at 3.5 dpf. Ectopic sprouts extending ventrally from the subintestinal vein are more common in mttpstl/stl mutant embryos than in mttpc655/c655 mutant embryos. Scale = 200 μM. (C) Quantification of the average number of ectopic sprouts in mttp mutants and siblings on 3.5 dpf. Results represent pooled data from 3 independent experiments, n = 28–36 total embryos/genotype group; mean +/- SD, Kruskall-Wallis with Dunn’s Multiple Comparison test, *** p < 0.001. (TIF) [file pgen.1008941.s013.tif]

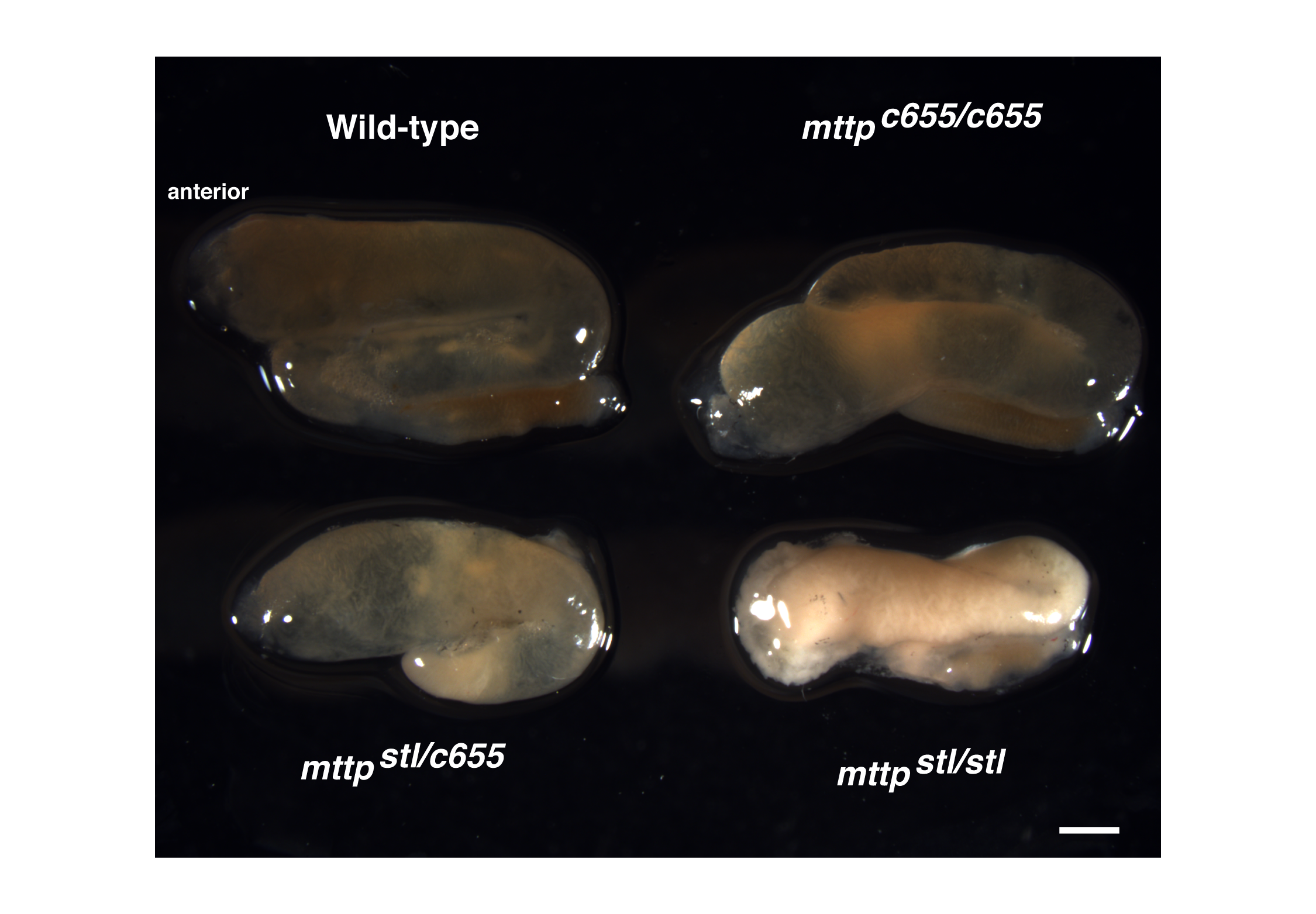

Supplement: S10 Fig — Representative images of isolated intestines from adult WT and mttp mutant fish (7.5 mo), scale = 1 mm. (TIF) [file pgen.1008941.s014.tif]

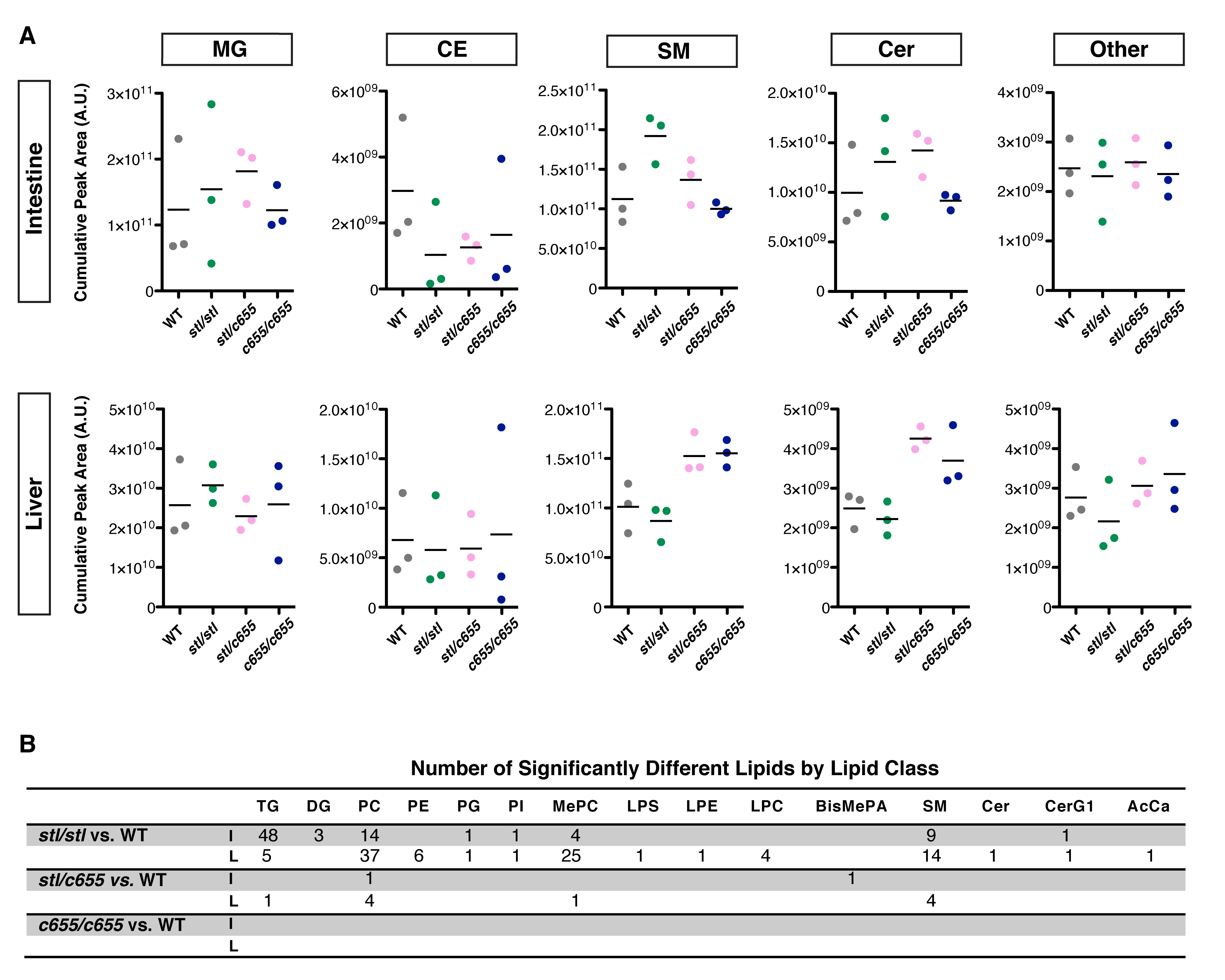

Supplement: S11 Fig — (A) Tissue lipid extracts from WT and mttp mutant lines were quantitated by LC-MS/MS and grouped into lipid classes and expressed as a sum of lipid group (n = 3). (B) The number of individual lipid species in the different lipid classes that are statistically different from WT in the intestine (I) or liver (L) (adjusted p-value < 0.20). Triacylglycerol (TG), diacylglycerol (DG), monoacylglycerol (MG), phosphatidylcholine (PC), phosphatidylethanolamine (PE), phosphatidylglycerol (PG), phosphatidylinositol (PI), methylphosphocholine (MePC), lysophosphatidylserine (LPS, lysophosphatidylethanolamine (LPE), lysophosphatidylcholine (LPC), bismethyl phosphatidic acid (BisMePA), sphingomyelin (SM), cholesterol ester (CE), ceramides (Cer), monoglycosylceramide (CerG1), acyl carnitine (AcCa). (TIF) [file pgen.1008941.s015.tif]

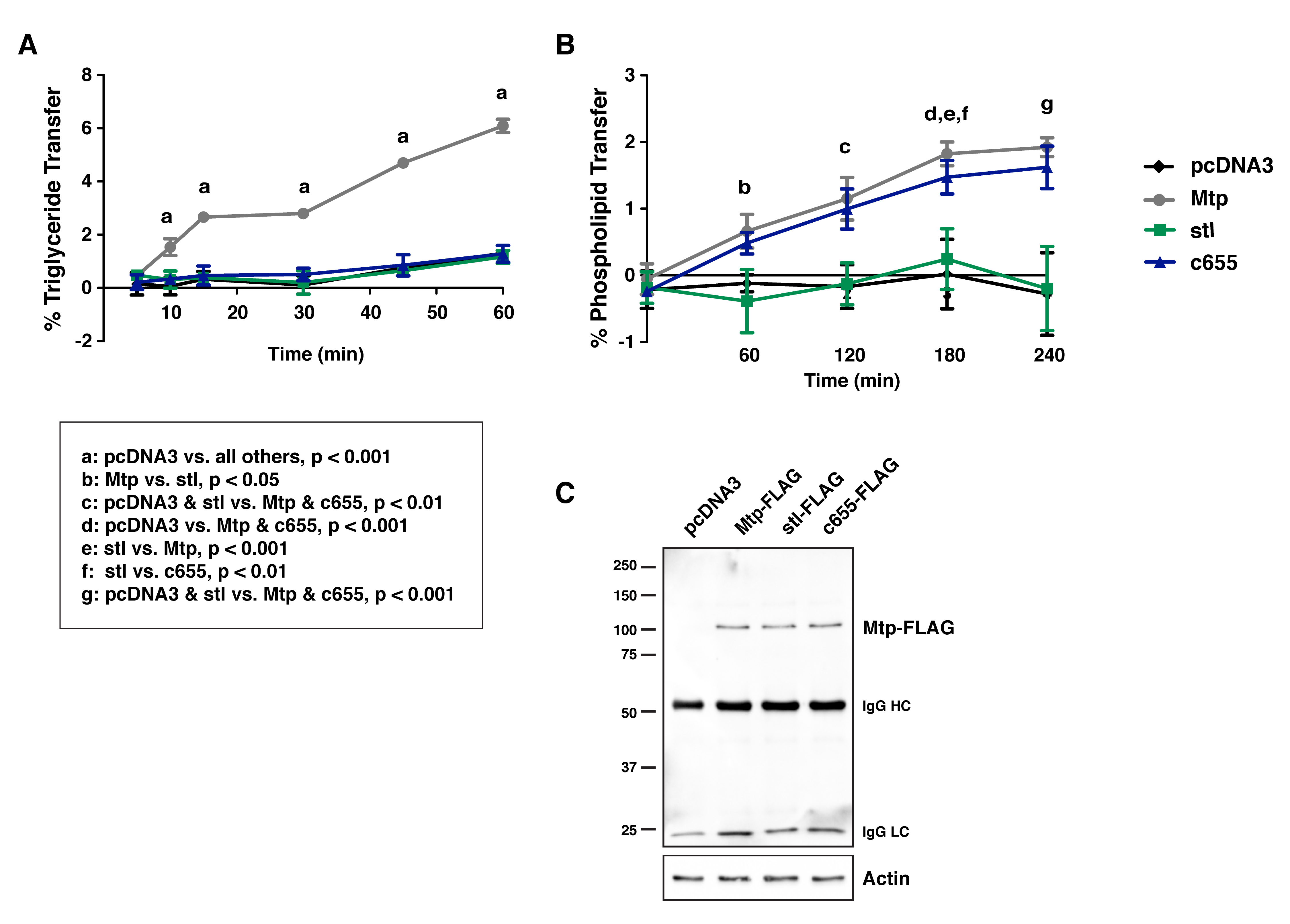

Supplement: S12 Fig — (A, B) Measurements for TG (A) and PL transfer (B) by zebrafish Mtp-FLAG and mutant proteins over a time-course. The single time-points depicted in the bar graphs of Fig 5E & 5F, correspond to the 45 min and 180 min (TG and PL transfer, respectively) time-points in the curves shown. For both, n = 3 (each n is the mean of three technical replicates from independent experiments), mean +/- SD, Repeated Measures ANOVA with Bonferroni post-hoc tests, significance as noted in figure. (C) Representative western blot of immunoprecipitated and eluted Mtp-FLAG proteins from COS-7 cell lysate used in the PL transfer assays. COS-7 cells transfected with FLAG-tagged proteins were immunoprecipitated from cell lysates using anti-FLAG antibodies and eluted with FLAG peptides. Blot on eluted fractions indicates equal concentrations of the various Mtp-FLAG proteins; actin blot indicates equal loading of cell lysate. (TIF) [file pgen.1008941.s016.tif]

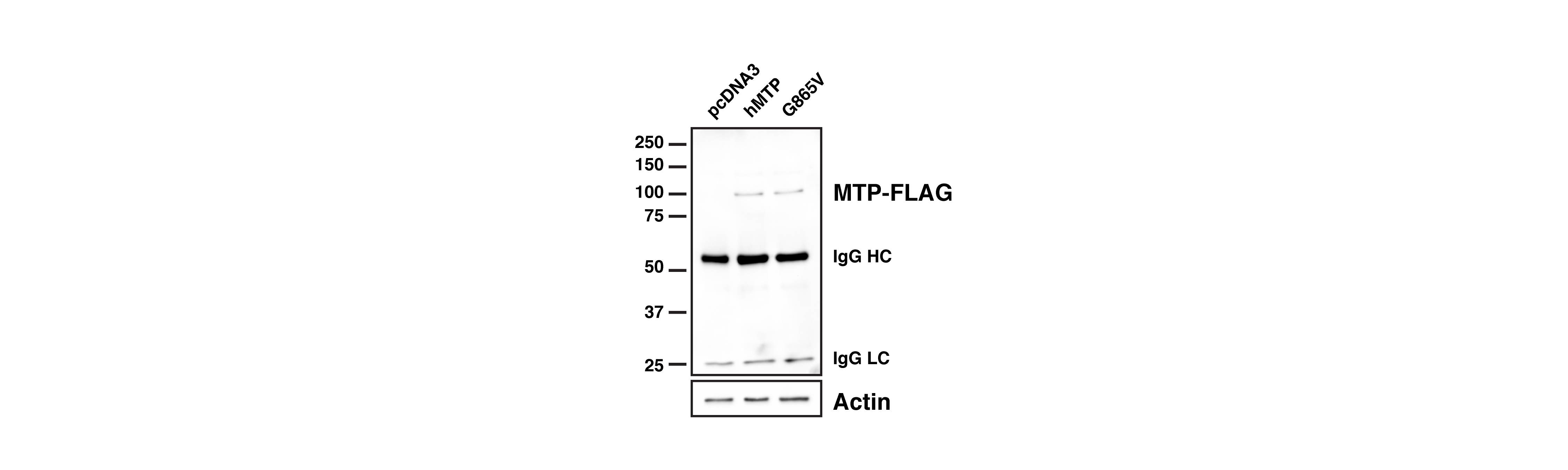

Supplement: S13 Fig — COS-7 cells were transfected and FLAG-tagged human MTTP proteins were immunoprecipitated from cell lysates using anti-FLAG antibodies and eluted with FLAG peptides. Representative western blot on eluted fractions indicates equal concentrations of the various MTP-FLAG proteins; actin blot indicates equal loading of cell lysate. (TIF) [file pgen.1008941.s017.tif]

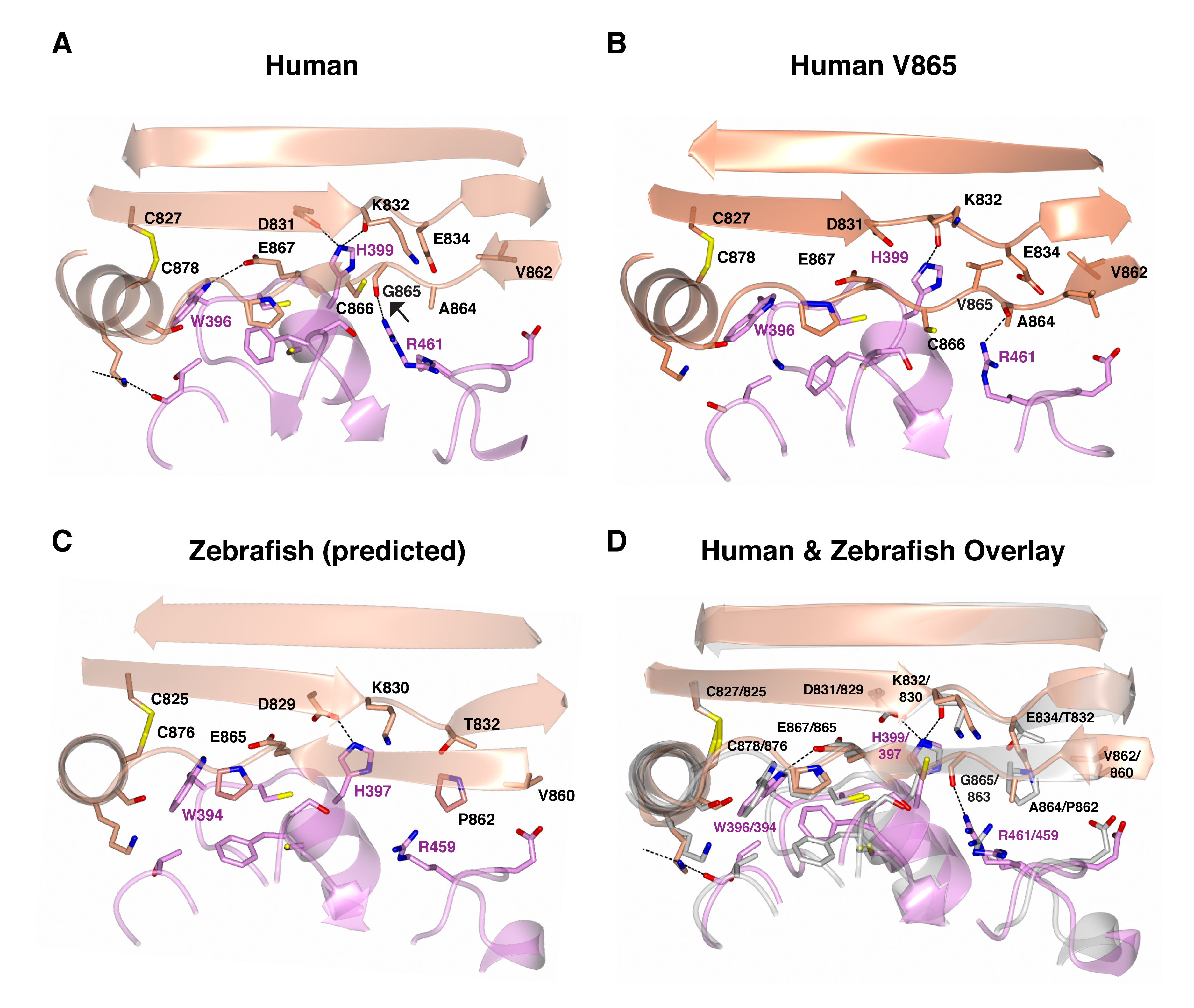

Supplement: S14 Fig — Views show the same region as in Fig 7E. (A) Wild-type human, (B) mutant V865 human, and (C) wild-type zebrafish shown separately. (D) Overlay of wild-type human and wild-type zebrafish. In (A), the backbone carbonyl of G865 is shown hydrogen-bonded to R461 of PDI (arrow). (TIF) [file pgen.1008941.s018.tif]
