# Supplementary material for: A point mutation decouples the lipid transfer activities of microsomal triglyceride transfer protein
Source: PLoS Genet. 2020 Aug 6;16(8):e1008941. doi: 10.1371/journal.pgen.1008941 (PMC7444587; doi:10.1371/journal.pgen.1008941)
Supplement: Attachment — Submitted filename: PLOS Genetics Response to reviewers 04102020.docx [file pgen.1008941.s019.docx]

**Reviewer #1:**

1. Does the mutation (zebrafish G863V or human G865V) alter the transfer of any other lipid besides triglyceride, e.g., cholesterol ester, sphingolipid, diacylglycerol?

We thank the Reviewer for raising this issue. In this study, we only measured TG and PE transfer activities. In the future, we plan to study the effect of this mutation on the transfer of other lipids such as cholesteryl ester, sphingolipid and diacylglycerol. Prior work has established that the phospholipid head group does not alter MTP transfer activity. We have added text explaining this in the Discussion section:

A putative second (PL) lipid-binding site was previously noted in the structure of the related protein, lamprey lipovitellin, at the junction between the N and A β-sheets (82). Future work will be needed to determine whether PL and other charged lipids may bind in an equivalent location between the N-terminal half β-barrel and the A-sheet of the C-terminal domain in MTP. In this study, we used PE as a substrate for the PL transfer assays. While prior work established that MTP PL transfer is not affected by the specific PL head group (21), we have not formally ruled out the possibility that the mutation confers some specificity for different phospholipid species. In future studies, we also want to further explore the G865V substrate transfer specificities beyond the two lipids used in this study (TG & PE).

2. p. 11, lines 293-296 – the authors write “this finding supports the biochemical evidence for an additional “slow” phospholipid transfer site (42) and provides additional evidence that this site is distinct from the lipid-binding cavity formed by the…..” The data are consistent with an additional phospholipid transfer site, but the authors do not present any data concerning the relative rate of transfer at that site. I would recommend removing the word “slow”.

The evidence for a “slow” phospholipid transfer was discussed by Atzel and Wetterau, 1994 in their biochemical studies. However, we do agree with the Reviewer that we do not present any evidence for the speed of phospholipid transfer, so we have removed the word slow. We have reorganized the text so that this topic is now covered in the Discusison section were we have added the following text:

The selective loss of TG transfer activity resulting from the G865V mutation is consistent with the hypothesis that the β-sandwich solely within the C-terminal domain predominantly transfers neutral lipids. This again argues that a second, distinct, binding site for PL must be present. Although this additional binding site is not clear from the crystal structure, the G865V mutation does not eliminate the activity of this site, suggesting that it may be located in a different region of the protein. A putative second (PL) lipid-binding site was previously noted in the structure of the related protein, lamprey lipovitellin, at the junction between the N and A β-sheets (82). Future work will be needed to determine whether PL and other charged lipids may bind in an equivalent location between the N-terminal half β-barrel and the A-sheet of the C-terminal domain in MTP.

3. Fig. 3 E, F – the authors do not comment on the results of the studies using the MTTP inhibitor lomitapide. It is interesting that lomitapide inhibits phospholipid transfer to the same degree in both the wild-type and mutant proteins suggesting it blocks transfer at both sites (if there are two). Some MTP inhibitors work presumably by blocking the lipid binding cavity in the C-terminal domain. Have you tried any other MTP inhibitors to see if you get the same result as you did with lomitapide? If you used an inhibitor that blocks solely at the lipid binding cavity, you might expect that the PL transfer activity in the G865V variant would not be affected. This doesn’t impact the conclusion that the phospholipid and triglyceride transfer can be decoupled; however, it does raise questions about the mechanism(s) of phospholipid transfer.

We too agree with the Reviewer that this topic is extremely interesting but beyond the scope of the current study. We have not yet tried other MTP inhibitors in this assay, however we are excited to perform these studies in future work. In particular, we are interested in testing the BMS-200150 compound we noted in the Discussion section (see excerpt below). We are also currently in the process of crystallizing MTP bound to lomitapide to determine where it binds and how it alters the structure of the protein complex. We agree that if we are able to obtain an inhibitor that specifically blocks the lipid binding cavity (without altering protein structure), it will help us to learn more about the mechanism of phospholipid transfer. Additionally, as discussed, if this putative inhibitor mimics the G865V variant, and phospholipid transfer is retained, it could potentially be a useful therapeutic.

The Discussion section includes the following:

While one of the original MTP inhibitors discovered, BMS-200150, was very effective at	inhibiting TG transfer, but less effective (~30%) at inhibiting PL transfer *in vitro* (86), later	studies on purified MTP protein indicated the compound inhibits transfer of both lipid	classes (94) and that it was not effective in animal models (85). Now that we appreciate	that the TG and PL transfer functions of MTTP can be dissociated, we argue that it may	be worth re-evaluating the PL transfer activity of any previously identified compounds	that inhibited TG transfer activity of MTP, but failed to inhibit APOB secretion *in vitro*.

4. Fig. 4 – the results of the experiments with the LipoGlo PAGE Gels suggest that the mttpc655/c655 embryos produce few VLDL particles; however, they do produce a significant number of particles in the LDL range. These particles must have core neutral lipid. If triglyceride transfer is blocked, what is in the core lipid in these particles? Cholesteryl ester? (This goes back to the question raised in point #1 above.)

Unfortunately, due to low yield of lipoproteins from the zebrafish, we have not been able to perform composition analyses on these particles. However, we have shown previously that *Drosophila* MTP (which lacks triglyceride transfer activity) synthesizes smaller TG-poor VLDL particles in the mouse liver (Khatun et al., Hepatology, 2012). At this time, it is unclear whether the LDL-sized particles in the zebrafish still contain triglyceride or cholesteryl ester in their core, and if so, how it is delivered to these particles. These questions will be more easily addressed in our future studies in which we are introducing the G865V mutation into the mouse.

5. Fig. 5 – There is a large difference in size of lipid droplets in the YSL of the mttpc655/c655 embryos compared to those in either the mttpstl/stl or the trans-heterozygotes. The authors speculate that retained neutral lipid is stored in larger lipid droplets in the YSL of mttpc655/c655 embryos because phospholipid is being secreted and consequently is less available to coat lipid droplets. Some basic arithmetic casts doubt on that hypothesis. A single droplet with a radius of 5 microns has the same surface area (phospholipid) as 25 droplets that have a radius of 1 micron. Consequently, it’s a bit of a stretch to say there is less phospholipid available in the mttpc655/c655 embryos without some quantitative data, which is beyond the scope of this investigation. The observation, nonetheless, is quite interesting and could be very important.

We agree with the Reviewer that this is an interesting phenotype and we have moved (and modified) this text from the Results to Discussion section.

It is intriguing to consider that the phenotypic differences in lipid droplet sizes within the YSL between the MTP mutant alleles may be due in part to the differing availability of PL. In the *c655* mutants it is conceivable that there is less PL available, potentially favoring larger lipid droplets that store more neutral lipid relative to the surface of the phospholipid coat. This hypothesis is consistent with data from a forward genetic screen in *Drosophila* S2 cells for modifiers of lipid droplet size and number wherein Guo et al. found that loss of genes critical for PL synthesis produced large lipid droplets [80]. In the current study, we have measured lipids only in adult organs; in future studies, we hope to develop methods to measure YSL lipid levels and directly test this hypothesis.

Minor comments:

• p. 2 – line 46 – “vassals” should be vessels. Corrected
• p. 2 – line 48 – why not say “humans” instead of “people”? Corrected
• p. 2, line 51 – why do you use the term “fats” for lipids? Is this to be a summary for the general public without scientific terminology? If it is not, why not say triglyceride? Yes, this is intended for a wide audience of scientists and non-scientists, although we eliminated the word “fats” anyway
• p. 3, line 81 – “ApoB” should be “APOB” by your convention. We have re-checked the whole manuscript for consistency in naming; these conventions are based on *1998 Trends in Genetics* "*Genetic Nomenclature Guide*"

• p.3, line 105 – there should be the word “on” between “based the” i.e., “based on the” Corrected
• p. 4, line 110 – “primary” should be “primarily” Corrected
• p. 18, line 473 – the word “present” should be “presents” Corrected

**Reviewer #2:**

1. Please take my criticism constructively. There are good aspects to the study, which in this reviewer’s mind need to be pulled out to reach a wider audience.

We appreciate the Reviewer’s appreciation of our study and their suggestions on how we could improve our manuscript.

2. Abstract-- The authors are assuming that the readership of PLOS genetics will know about Mttp-and there is no reason why they should. Really not a good Abstract to sell your story-only people with specialist knowledge/interest would be encouraged to read this manuscript. You are under-selling your work. What is the unique selling point of your study design? What is it a good example of?

We have taken these suggestions quite seriously. The new abstract provides more background so a wider array of scientists will appreciate the novelty and importance of the study. For example, we added the following sentences to the abstract:

Zebrafish embryos produce large numbers of B-lps in the yolk syncytial layer (YSL) to move lipids from yolk to growing tissues. Disruptions in B-lp production perturb yolk morphology, readily allowing for visual identification of mutants with altered B-lp metabolism. Here we report the discovery of a missense mutation in microsomal triglyceride transfer protein (Mtp), a protein that is essential for B-lp production. This mutation, a conserved glycine residue to valine (zebrafish G863V, human G865V), reduces B-lp production, and results in yolk opacity due to aberrant accumulation of cytoplasmic lipid droplets in the YSL.

3. Introduction-- I feel that the authors haven’t provided the Introduction in terms of why they did what they did. In contrast to most papers (which put the Introduction in the Discussion), you have put the Discussion in the Introduction, perhaps? The Introduction does not set you up for what you have done. Why, did you start from the point you have? What was your motivation? Almost the first sentence of the Results is the first sentence of the Introduction? Why were you searching zebrafish for defects in yolk utilization-why was this important to do?

We have now re-organized the manuscript and added more information about yolk biology and why we were looking for zebrafish mutants with opaque yolks. We hope that the manuscript is now clear and more accessible to a wider audience.

4. Introduction-- You talk a great deal about PDI in the results and yet this is not mentioned.

We have addressed this concern in the following paragraph of the Introduction:

MTP is a heterodimer of the large M or MTPα subunit (~97-kDa, encoded by the *MTTP* gene) and the smaller P or MTPβ subunit, protein disulfide isomerase (PDI; ~58-kDa human subunit) (15, 16). Vertebrate MTP can bind and transfer triacylglycerol, diacylglycerol, phospholipid, cholesteryl ester, ceramide, and sphingomyelin between vesicles *in vitro* (17-22). Kinetic studies suggest that MTP transiently interacts with membranes, acquires lipids, and then delivers these lipids to an acceptor membrane. The transfer of lipids occurs down a concentration gradient and does not require energy (23, 24).

5. Results-- Lack of apoB secretion-phraseology correct (sic) but for readers of Plos Genetics and the phenotype you are seeing would come across better if you link it up with a defect in lipid secretion, what is the evidence here that the lipid is only being secreted on ApoB-lipoproteins-again given the conclusion you are drawing-perhaps the Introduction should remind us what the chief lipids in the yolk sac of fish are? And how they are exported out of the yolk. Do fish have VTG?

By including more information on yolk biology, including the types of lipids present in the	zebrafish yolk, in the introduction, we hope that it is clear that lipids are being secreted	from the YSL via lipoproteins. There is currently no evidence that yolk lipids are being	secreted from the YSL via any alternative route. The following paragraph was added to	the Introduction:

In zebrafish and other lecithotrophic teleosts, B-lps are essential during embryonic development to transport lipids from the yolk to the developing tissues before the larvae commence feeding (33-36). Yolk proteins and lipids are deposited into oocytes by way of vitellogenin, a specialized type of high-density lipoprotein synthesized by the maternal liver (37). Once in the developing oocyte, vitellogenin undergoes proteolytic cleavage to phosvitin and lipovitellin and, in the case of zebrafish, the lipid is stored in yolk granules/platelets (38, 39). In zebrafish, the yolk contains many different lipid classes, with the most abundant being cholesterol, phosphatidylcholine, triacylglycerol, phosphatidylinositol, phosphatidylethanolamine, diacylglycerol, cholesteryl esters, and sphingomyelins (40). During embryogenesis, the lipids in the yolk platelets undergo lipolysis and re-esterification and are then packaged into lipoproteins in the ER of the yolk syncytial layer (YSL), a multi-nucleated cytoplasm that surrounds the yolk mass (41-44). The zebrafish YSL expresses both ApoB and MTP, and produces large numbers of B-lps (34, 43, 45-48). These lipoproteins are secreted into the circulation and provide energy and building blocks for the developing embryo.

6. Have you checked the lipid composition of the egg-yolk in the mutants? This is sort of addressed in Figure 5 but even so.

We did not directly check the lipid composition of the yolk in the mutant fish. These lipids are synthesized and packaged into vitellogenin in the liver of the mother before being taken up by developing oocytes. We were crossing fish that were heterozygous for the mutations for our experiments, and since we did not see any differences in the embryos or adults of heterozygous fish vs. wild-type, we do not expect any changes in the lipids that are deposited into the yolks of the mutants.

7. Bottom of Page 7/top of 8, expecting to see the result before new section heading-perhaps, they need to remove to under the next section heading.

The manuscript has been re-organized and we have included additional headings throughout.

8. I would liked to have been told what Lomitapide was developed to inhibit and given the structure of MTTP where it is envisaged to bind.

We agree with the Reviewer and added the following to the Discussion section:

Inhibition of MTP has long been considered a possible therapeutic target for lowering disease risk by inhibiting the production of VLDL and chylomicrons (85, 86) (for review see (30, 87)). Currently, the only MTP inhibitor approved for use in patients is lomitapide (Juxtapid®, BMS-201038), which is a derivative of a compound discovered in a high-throughput chemical library screen specifically for MTP inhibitors (85, 86). Although its binding site is not known, lomitapide inhibits both TG and PL transfer and reduces APOB secretion (88) (Fig 6E,F).

9. I think with a better Introduction, you could present your results in a more logical order. What is the evidence for the statement ‘During this time, the fish are relying solely on yolk lipids’ Also what is the evidence for the next statement, “Until 5 days post fertilization, the fish are unable to eat exogenous food and rely solely on yolk nutrients.”

Here to we agree with the Reviewer and reordered the Results section. We have now added appropriate references and made clarifications in the text. We also added the following text to the Introduction section:

In zebrafish and other lecithotrophic teleosts, B-lps are essential during embryonic development to transport lipids from the yolk to the developing tissues before the larvae commence feeding (33-36).

10. The phospholipid transfer activity based on just one phospholipid species-is it the most suitable one.

In prior work we established the MTP transfer activity assay and specifically explored its abilities to transfer a variety of lipids to different acceptor lipid particles (<https://www.jlr.org/content/45/4/764.long>). We then specifically improved the assay to characterize phospholipid transfer by incubating different amounts of MTP with donor vesicles containing fluorescent PE ( <https://www.jlr.org/content/46/8/1779.long>). It has been established by Jamil et al., 1995 that the phospholipid head group does not alter the transfer activity: “Additional modifications to the head group resulting in zwitterionic PE, or negatively charged PS, PA, and PI, did not change the transfer rate significantly from that of PC.” <https://www.jbc.org/content/270/12/6549.full>.

We modified the discussion, see Reviewer 1 Point 1

11. How do you know the MTTP:PDI complex is binding triglyceride?

We thank the reviewer for the question and the opportunity for us to clarify. We have addressed this point with clarification of the following text in the introduction.

MTP is a heterodimer of the large M or MTPα subunit (~97-kDa, encoded by the *MTTP* gene) and the smaller P or MTPβ subunit, protein disulfide isomerase (PDI; ~58-kDa human subunit) (15, 16). Vertebrate MTP can bind and transfer triacylglycerol, diacylglycerol, phospholipid, cholesteryl ester, ceramide, and sphingomyelin between vesicles *in vitro* (17-22).

While we did not directly test whether the MTTP:PDI complex carrying the G865V mutation is unable to bind triglyceride, our modeling data suggests that the mutation would block access to the lipid binding site, thus inhibiting binding of TG. We have clarified this point in the text of the Discussion section.

The G865V mutation would alter the M subunit-PDI interaction, which we hypothesize	may prevent movement of the **a’** domain and block access to the lipid-binding site,	thereby attenuating TG binding and transfer (and perhaps transfer of other neutral	lipids).

12. A lot of data but doesn’t quite grab this reviewers attention-because a clear story is not coming through. Just wonder whether the problem started from the Introduction-I feel you are not really getting across why the study is important-you present some of the key results at the beginning and then things rather fade away. The novelty of your apoB assay could come earlier to grab our attention, perhaps. I think if you reduced the description of your results by at least 20%, you would get the main points across more effectively. I would encourage you have more regular subheadings: in some cases they could say more.

We greatly appreciated the Reviewer’s constructive suggestions and have sharpened the abstract, reorganized the Introduction, changed the order of the data presentation and added subheadings.
