# Supplementary material for: A point mutation decouples the lipid transfer activities of microsomal triglyceride transfer protein
Source: PLoS Genet. 2020 Aug 6;16(8):e1008941. doi: 10.1371/journal.pgen.1008941 (PMC7444587; doi:10.1371/journal.pgen.1008941)
Supplement: Attachment — Submitted filename: PLOS Genetics Response to reviewers 06112020 .docx [file pgen.1008941.s020.docx]

Reviewer #1: “Thank you for the thoughtful responses to my initial comments.”

We appreciate the Reviewer’s kinds words and acknowledgement of how seriously we have taken the Reviewers’ comments

Reviewer #2:

1) Abstract …Much Improved

We appreciate that the Reviewer recognized that we not only added new data but took the extra time to rewrite the entire manuscript beyond just the abstract. We took Reviewer 2’s organizational suggestions to heart in the revision.

2) Results: Could you please tell the reader the nature of the other MTTP variant found (predicted to be tolerated in Table S1)

We have added the following to the legend for Figure 1:

“An additional SNV in *mttp* at position Chr1:11,421,300 GRCz10 (T/C) causing a missense mutation (M850T) was also identified in *c655* mutants, however, this SNV was not predicted to be deleterious and has been previously noted in Ensembl.”

3). Results: Figure 4, Second paragraph in Section c665 mutant adults are largely protected from Intestinal steatosis is still a problem. I became rather bored here (all rather woolly) until I looked at the Figure and then realised (sic) the key aspects of the data are not spelt out for the reader. I feel the author spent too much time justifying what they did (First two sentences) rather than getting to the point and focusing in on the lipidomic analysis, which is key to linking into the next section. The data appear equivocal (potentially due to small sample sizes) but I think the authors data in the liver on the TG and DG in their c655 mice are consistent with their hypothesis. What is not different is as potentially important result as was is potentially different. Would be good to corroborate TG/DG data on the yolk.

We are puzzled at which result the reviewer considers equivocal. The body size and histological findings are dramatic, as is a 3-fold increase in triglyceride levels that is reproducible across 3 replicates. While we concede that other aspects of the lipidomic data may be more complex and nuanced, the TG accumulation in the intestine of stl mutants is irrefutable, and is thus the crux of our ensuing discussion. Further, if the reviewer finds any aspect of this panel equivocal, they should be encouraged by the numerous independent lines of evidence and orthogonal tests that are used to support the same conclusion (body weight/size, histology, lipophilic staining, electron microscopy, and lipoprotein profiling all support that c665 mutant adults are largely protected from Intestinal steatosis). Thus we believe the introductory sentences do serve an essential function in that they prime readers to interpret these new findings in the context of the previous findings established in younger animals.

We are saddened that the Reviewer found parts of this section “woolly” or “boring” as we and many of our colleagues found these results quite exciting and compelling. Irrespective of its apparently variable level of intrigue for different readers, we believe that authors and reviewers would agree that this section represents a valid finding that is useful for the interpretation of future results and the broader conclusions of the paper, and should thus remain in the main text as written.

4). On page 14, you say you were surprised to find that the c655-surely that was what you were expecting from the lipidomic analysis & lipoprotein analysis? Myself, I would be reassured rather than surprised by this result-corroborating the arguably weak lipidomic data. You are not quite making the link with the lipidomic data, the results in this section are almost backwards. The PDI aspect is interesting but perhaps best after you tie up the lipid aspects. Then in would be easier to link up the PDI story with the structural data. Manuscript still too turgid in places for this impatient reviewer.

We have clarified the main text to emphasize the root of our surprise as quoted below. While we obviously expected c655 to be a hypomorphic allele, none of the many previously identified mutations in mttp have dissociated TG and PL transfer activities. This is one of the key novelties of this manuscript and was quite surprising. The sentence now reads:

*“However, we were surprised to find that unlike all previously identified hypomorphic MTP alleles, the c655 mutation has differential effects on the transfer activities of different lipid species.”*

We do not believe that detailed discussion of these *in vitro* findings in the context of the lipidomic data is warranted in this section of the results. Further, it is difficult for us to understand how the lipidomic data (which shows little or no difference in the lipid profiles in c655 mutants) could be used to corroborate or foresee the *in vitro* results.

Finally, we apologize if parts of the manuscripts seem turgid. Many adjustments have already been made at the suggestion of reviewers, and it does not seem appropriate to retroactively remove them at this stage of review.

5). I think you send too much time on Fig 6- could almost be Supplementary Figure-just say it recapitulates the zebrafish data and point out significance of inhibitor.

Here we strongly disagree with the Reviewer. Showing that the fish data aligns nicely with human data remains an important finding. This Reviewer may be aware of the close alignment between fish, mouse, and human biochemical and molecular studies, but sadly many biologists still question the relevance of zebrafish studies on human disease. These data are just one more example of the power of the zebrafish system.

6) I think Discussion could be shorter-and rejigging the presentation of your data.

We appreciate the Reviewer’s suggestion, and after a careful review of the discussion section have shortened the section as outlined below (the following was deleted):

- “…including reflux, indigestion, abdominal pain, constipation, and diarrhea”

However, we are reticent to perform further “rejigging” of the discussion section, which has already been substantially reorganized throughout the review process and has met the high standards set by multiple reviewers and the authors themselves. We believe further modifications at this stage would detract from the many improvements attributable to review process so far.
